# Suturing Versus Adhesion for Mesh Fixation in Ventral Hernia Repair and Abdominal Wall Reconstruction: A Systematic Review and Network Meta-Analysis

**DOI:** 10.7759/cureus.51535

**Published:** 2024-01-02

**Authors:** Jimmy Mena, Elia Azir, Rizwan Ahmed, Mohammad Ali, Michael Adesida

**Affiliations:** 1 General Surgery, Imperial College London, London, GBR; 2 General Surgery, Princess Royal Univeristy Hospital, London, GBR; 3 General Surgery, Princess Royal University Hospital, London, GBR

**Keywords:** abdomen ventral hernia, tissue adhesive, abdominal wall reconsruction, mesh fixation, mesh repair

## Abstract

Abdominal wall reconstruction (AWR) is a surgical procedure performed to address various conditions such as hernias, incisional hernias, and complex abdominal wall defects. Mesh fixation plays a crucial role in providing mechanical reinforcement to the weakened abdominal wall during AWR. Traditionally, suturing has been the preferred method for mesh fixation; however, adhesion techniques using tissue adhesives or glues have gained attention as an alternative approach. This systematic review aims to compare suturing and adhesion techniques for mesh fixation in AWR and assess their effectiveness in preventing hernia recurrence.

A comprehensive literature search was conducted across relevant databases, including PubMed, MEDLINE, Embase, and the Cochrane Library. Studies that fulfilled the predetermined eligibility criteria were included. The primary outcome measure of interest was hernia recurrence rates. Secondary outcomes included mesh-related complications, surgical site infections, patient-reported outcomes, and functional outcomes. A risk of bias assessment was performed for the included studies, and data were synthesized qualitatively.

Overall, the results of the included studies suggest that atraumatic mesh fixation with glue may have the potential to reduce chronic groin pain (CGP). However, there were significant variations in patient selection criteria, glue administration techniques, and hernia repair methods among the trials, which limited the ability to draw definitive conclusions. Additionally, the definitions of CGP and measurement scales for postoperative pain varied across studies, making it challenging to compare outcomes.

The limitations of the review include the small sample sizes in some trials, relatively short follow-up durations, and the lack of standardized criteria for assessing variables such as foreign body sensation and groin compliance. Furthermore, the economic implications of using glue fixation compared to traditional suture fixation need to be considered.

## Introduction and background

There are multiple classification guidance for hernias in literature. Hernias can be classified according to site, reducibility, and onset (congenital or acquired). Anatomically hernias can be mainly classified into Ventral hernias and Groin hernias.

Ventral hernias can be further classified into Epigastric (occur due to a weakness or opening in the muscles of the upper abdominal wall), Umbilical (occur near the bellybutton that has a natural weakness from the blood vessels of the umbilical cord), Lumbar (a rare type of hernia that occur through defects in the lumbar muscles or the posterior fascia, below the 12th rib and above the iliac crest), Spigelian (occur through a weakness between the muscle fibers of the abdominal wall. It develops between the muscles of the abdominal wall rather than protruding through layers of fat. This is quite a rare type of hernia which occurs in both males and females. It often develops in later life when the abdominal muscles have become weaker), and Incisional (hernias that appear in the abdomen at the site of previous surgery. These can vary in size from small to very large and complex).

Groin hernias are located in the lower abdomen just above the inguinal ligament, near or adjacent to the pubic area. Inguinal and femoral are the two types of groin hernias and can produce pain that extends into the upper thigh or scrotum. Inguinal hernias can be classified as “direct” or “indirect”. An indirect inguinal hernia occurs through the natural weakness in the internal inguinal ring and a direct inguinal hernia is a result of weakness in the floor of the inguinal canal and is more likely to develop in older men over the age of 40. The floor of the inguinal canal is located just below the internal inguinal ring.

Abdominal wall reconstruction (AWR) is a surgical procedure performed to address a variety of conditions, including hernias, incisional hernias, and complex abdominal wall defects. These conditions can result from trauma, infection, previous surgeries, or congenital anomalies. AWR aims to restore the integrity and functionality of the abdominal wall, improve patient quality of life, and prevent complications such as recurrence or bulging [1]. A critical aspect of AWR is the fixation of a mesh prosthesis, which provides mechanical reinforcement to the weakened abdominal wall. The proper fixation technique plays a crucial role in achieving long-term success by promoting mesh integration, reducing complications, and enhancing patient outcomes.

Currently, two main methods are utilized for mesh fixation: suturing and the use of adhesives [2]. The history of suturing for mesh fixation in AWR can be traced back to the pioneering work of surgeons in the field of hernia repair. The use of prosthetic materials for hernia repair has evolved over centuries, with early attempts involving the use of natural materials such as silk or animal-derived tissues. However, these materials were associated with high rates of infection and poor long-term outcomes [3].

In the 1950s, the introduction of synthetic materials, such as polypropylene mesh, revolutionized hernia repair. Surgeons began incorporating these mesh prostheses to reinforce the weakened abdominal wall and improve surgical outcomes. Initially, sutures were used to directly anchor the mesh to the abdominal wall. However, the recognition of the importance of tension-free repairs led to the development of new techniques and approaches [4]. In the 1980s, the concept of "sublay" repair gained popularity. This technique involved placing the mesh in a space created between the abdominal wall layers, such as the preperitoneal or retrorectus space. The mesh was then secured using sutures or other fixation methods. This approach allowed for better tissue coverage, reduced tension on the abdominal wall, and improved outcomes [5].

Over time, advancements in suture materials, surgical techniques, and mesh designs further refined the technique of suturing for mesh fixation in AWR. The emphasis on minimizing complications, optimizing tissue integration, and achieving durable repairs has led to ongoing research and innovation in the field [6]. In recent years, the use of minimally invasive techniques, such as laparoscopic or robotic-assisted approaches, has also influenced the technique of suturing for mesh fixation. These techniques offer the advantage of smaller incisions, reduced trauma to the abdominal wall, and potentially faster recovery. Suturing techniques have been adapted and refined for use in these minimally invasive procedures, allowing for precise mesh placement and fixation [7]. The technique of suturing for mesh fixation in AWR has a long history and has evolved over time. The use of sutures to secure prosthetic materials for hernia repair dates back several decades. Early techniques involved the use of non-absorbable sutures, such as nylon or polypropylene, to anchor the mesh to the abdominal wall. However, the emphasis on tension-free repairs gained prominence with the recognition that tension on the abdominal wall increases the risk of recurrence and complications [8].

With the advent of tension-free repairs, the technique of suturing for mesh fixation underwent significant advancements. Instead of placing sutures directly through the mesh, the focus shifted to securing the mesh in a "sublay" position, i.e., placing it in the preperitoneal or retrorectus space. This approach allowed for better tissue coverage and reduced the risk of mesh-related complications. Various suture materials, including absorbable and non-absorbable sutures, have been utilized in AWR, and different suture configurations, such as interrupted or continuous sutures, have been employed based on surgeon preference and patient factors [9]. AWR is a surgical procedure performed to address conditions such as hernias, incisional hernias, and complex abdominal wall defects resulting from various factors, including trauma, infection, previous surgeries, or congenital anomalies. The primary goal of AWR is to restore the integrity and functionality of the abdominal wall, improve patient quality of life, and prevent complications [10].

Mesh fixation plays a crucial role in AWR by providing mechanical reinforcement to the weakened abdominal wall. Traditionally, suturing has been the preferred method for mesh fixation. However, the use of adhesives as an alternative technique has gained attention in recent years. Adhesives offer potential benefits such as reduced surgical time, ease of application, and potentially decreased postoperative pain compared to sutures.

Tissue adhesives or glues used for mesh fixation can be classified into synthetic glues, such as cyanoacrylate, or biological glues derived from human or animal sources. Synthetic glues are often based on cyanoacrylate compounds, which polymerize rapidly upon contact with tissue fluid, forming a strong adhesive bond. Biological glues, such as fibrin sealants, are derived from blood components and mimic the natural clotting process to secure the mesh [11]. The use of tissue adhesives in surgery, including mesh fixation, has a long history. The development of synthetic tissue adhesives began in the 1940s, with the introduction of cyanoacrylate compounds. These compounds demonstrated excellent adhesive properties and were initially used in wound closure and skin approximation. However, concerns about tissue toxicity and inflammation limited their widespread use [12].

Over time, advancements in adhesive technology and improved understanding of tissue response led to the exploration of adhesives for mesh fixation in AWR. The potential advantages of adhesives, such as reduced operative time, elimination of the need for sutures, and potentially lower rates of chronic pain, have attracted interest from surgeons [13]. In recent years, research efforts have focused on evaluating the efficacy, safety, and long-term outcomes of adhesion for mesh fixation in AWR. Studies have investigated various adhesive materials, techniques, and application methods. Comparisons between adhesive fixation and traditional suturing techniques have been explored to determine the relative merits and limitations of each approach [14].

Challenges associated with adhesion techniques include concerns regarding adhesive strength, mesh displacement or detachment, and potential long-term complications such as adhesions or seromas. Optimizing adhesive properties, understanding the impact of different adhesive materials on tissue response, and developing standardized techniques for mesh fixation are ongoing areas of research [15]. The choice between suturing and adhesion for mesh fixation in AWR remains a subject of debate among surgeons. While both techniques have been utilized in clinical practice, their comparative effectiveness and associated complications have not been comprehensively studied and synthesized.

This systematic review aims to provide a scientific background by critically analyzing the available literature to address the following research questions: What is the comparative effectiveness of suturing versus adhesion for mesh fixation in AWR? What are the short-term and long-term outcomes associated with suturing and adhesion techniques? What are the complications and adverse events associated with suturing versus adhesion? Are there specific patient populations or clinical scenarios where one technique may be preferred over the other?

By conducting a systematic review of the existing literature, we aim to identify and analyze relevant studies, including randomized controlled trials (RCTs), cohort studies, and case series, to evaluate the comparative effectiveness, safety, and patient outcomes associated with suturing and adhesion techniques for mesh fixation in AWR.

## Review

Methodology

We performed this systematic review and meta-analysis in accordance with the recommendations of the Preferred Reporting Items for Systematic Reviews and Meta-Analyses (PRISMA) and Meta-analyses of Observational Studies in Epidemiology (MOOSE) statements. PRISMA and MOOSE are reporting checklists for authors, editors, and reviewers of meta-analyses of interventional and observational studies. According to the International Committee of Medical Journal Association (ICJME), reviewers must report their findings according to each of the items listed in those checklists.

Search Strategy and Screening

An electronic search was conducted from the inception till May 2023 in the following bibliographic databases: Medline via PubMed, Scopus, Web of Science, and Cochrane Central Register of Controlled Trials (CENTRAL).

used different combinations of the following queries: ("abdominal wall reconstruction" OR "ventral hernia repair") AND ("suturing" OR "suture fixation") AND ("mesh" OR "prosthesis") AND ("adhesion" OR "tissue adhesive" OR "glue"). The authors then independently excluded non-relevant articles based on the review of the full-text articles before comparing, and the selected publications reporting on outcomes of patients with any abdominal wall reconstruction published in the English language were included. Upon uncertainty of inclusion of a publication an additional author was consulted.

The primary outcome measure for articles included in the study "Suturing versus Adhesion for Mesh Fixation in Abdominal Wall Reconstruction" was the comparison of hernia recurrence rates between suturing and adhesion techniques for mesh fixation in abdominal wall reconstruction. Hernia recurrence is a critical outcome in this context as it reflects the long-term success of the surgical repair and the durability of the mesh fixation method. The articles were assessed based on their ability to provide data on hernia recurrence rates, which were evaluated through clinical follow-up, imaging studies, or patient-reported outcomes. By focusing on hernia recurrence as the primary outcome measure, the study aimed to determine the effectiveness and comparative efficacy of suturing and adhesion techniques in achieving durable mesh fixation and preventing hernia recurrence in abdominal wall reconstruction.

Retrieved citations were imported into EndNote X7 (Clarivate Plc, Philadelphia, PA) for duplicate removal. Subsequently, unique citations were imported into an Excel sheet (Microsoft, Redmond, WA) and screened by two independent reviewers; the screening was conducted in two steps: title and abstract screening, followed by a full-text screening of potentially eligible records.

Data Extraction

Data entry and processing were carried out using a standardized Excel sheet and reviewers extracted the data from the included studies. The extracted data included the following domains: (1) Summary characteristics of the included studies; (2) Baseline characteristics of studied populations; and (3) Study outcomes. All reviewers independently extracted data from the included articles and any discrepancies were solved by discussion.

Dealing With Missing Data

Missing standard deviation (SD) of mean change from baseline was calculated from standard error or 95% confidence interval (CI), according to Altman.

Statistical Analysis

Data were fed to the computer and analyzed using SPSS software, version 20.0 (IBM Corp., Armonk, NY). The Kolmogorov-Smirnov was used to verify the normality of the distribution of variables, a paired t-test was used to compare two periods for normally distributed quantitative variables while ANOVA with repeated measures was used for comparing the different studied periods for normally distributed quantitative variables and followed by Post Hoc test (Bonferroni adjusted) for pairwise comparison. Pearson coefficient was used to correlate between two normally distributed quantitative variables. The significance of the obtained results was judged at the 5% level.

Assessment of Heterogeneity

We assessed heterogeneity by visual inspection of the forest plots, Chi-square, and I-square tests. According to the recommendations of the Cochrane Handbook of Systematic Reviews and Meta-Analysis, a Chi-square p-value less than 0.1 denotes significant heterogeneity while I-square values show no important heterogeneity between 0% and 40%, moderate heterogeneity from 40% to 60%, substantial heterogeneity from 60% to 100.

Results

Study Selection

**Figure 1:**
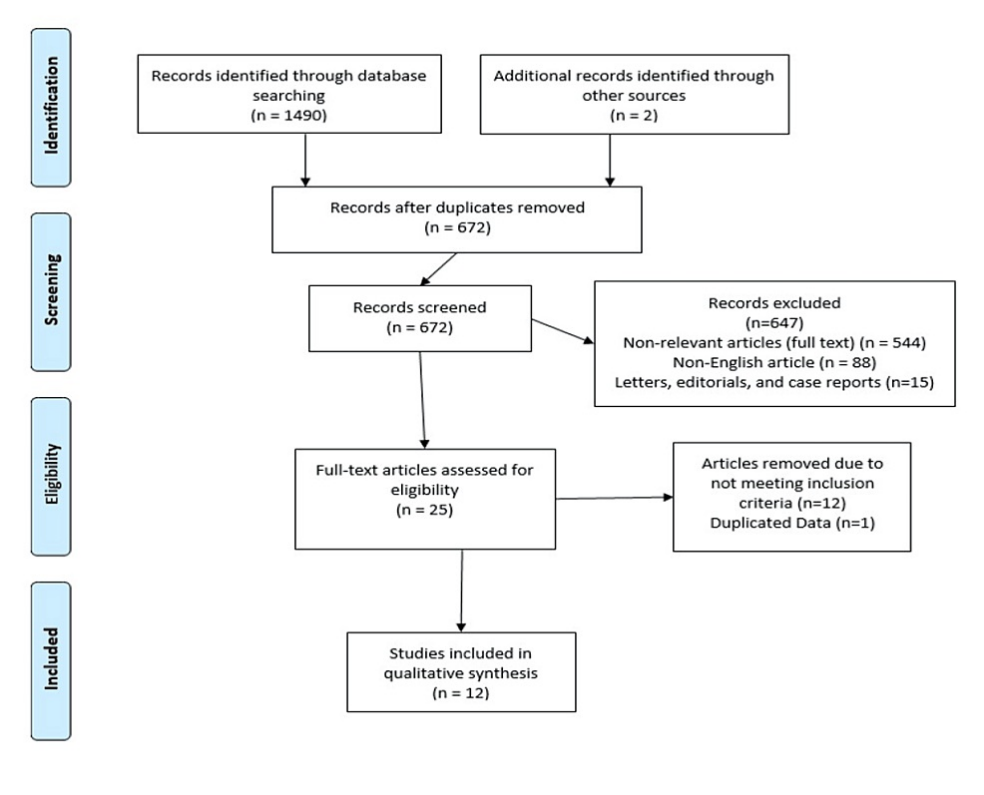
PRISMA flow chart for systematic review PRISMA: Preferred Reporting Items for Systematic Reviews and Meta-Analyses

In the present study, we searched the following electronic databases for published articles from January 1990 to May 2023: Embase, Scopus, PubMed, Google Scholar, Cochrane Library, and ClinicalTrials.gov, using appropriate combinations of keywords. The literature search resulted in 1490 studies. After removing duplicates, a total of 672 studies were identified. After the screening of abstracts, 647 studies did not meet the criteria and were excluded (544 were non-relevant articles, 88 were non-English articles and 15 were either letters, editorials, or case reports making these irrelevant to this analysis). Twelve articles were found eligible for inclusion and one had duplicated data. So, 12 studies (total no. of patients = 309) were included in the present systematic review (Figure 1). Overall, the studies showed high quality within the limits of their design.

An overview of the reported results from the included studies is summarized in Table 1.

**Table 1:** Demographic data in patients with mesh fixation in abdominal wall reconstruction

Study	Type of surgery	Sample size	Age	Gender	
				Male	Female
Nowobilski et al., 2004	sutures	24	52.6	46	0
	adhesive	22	60.5		
Hidalgo et al., 2005	sutures	55	49-71	55	0
	adhesive				
Testini et al., 2010	sutures	59	58	16	
	adhesive	106			
Wong et al., 2011	sutures	26	55.19	20	
	adhesive	30	55.9		
Kim-Fuchs et al., 2011	sutures	133	56.8	264	0
	adhesive	131	55.1		
Negro et al., 2011	sutures	171	55	159	16
	adhesive	349	55	325	24
Paajanen et al., 2011	sutures	151	53	135	16
	adhesive	151	53	131	20
Giampiero et al., 2011	sutures	158	59	158	0
	adhesive	158	58	158	0
Jani K, 2012	sutures	127	44.8	119	8
	adhesive	124	47.2	115	9
Jeyakumar et al., 2018	sutures	19	31-60	-	-
	adhesive	17			
Shukla et al., 2019	sutures	60	45.95	-	-
	adhesive	60	47.9		
Arash et al., 2020	sutures	58	53.1	58	-
	adhesive		55.8		

Outcome Characteristics

In a study by Nowobilski et al. (2004), the use of sutures for hernia repair was investigated in 24 participants with a mean age of 52.6 years. The follow-up duration was 4.7 months. Among the participants, 33% had direct hernias. The mean operation time was 42.1 minutes, and the pain score was reported as 5. No complications were reported, and the recurrence rate was 5%. The mean hospital stay was 1.75 days. Additionally, the study examined adhesive mesh fixation in 22 participants with a mean age of 60.5 years. The follow-up duration was 4.1 months, and 36% of participants had direct hernias. The mean operation time was 40.2 minutes, and the pain score was reported as 4.1. No complications were reported, and the recurrence rate was 8%. The mean hospital stay was 1.25 days [16].

Hidalgo et al. (2005) conducted a study involving 55 male participants with an age range of 49-71 years. The follow-up duration was 12 months, and the focus was on bilateral inguinal hernias. No significant differences were found in hernia classification. However, specific details regarding operation time, pain score, complications, recurrence, and hospital stay were not reported. The study did not provide information on the use of adhesive mesh fixation [17].

Testini et al. (2010) conducted a study on sutures and adhesive mesh fixation for unilateral inguinal hernias. The study included 59 participants with a mean age of 58 years, but no gender distribution was specified. The follow-up duration ranged from 14 to 60 months. However, specific details regarding hernia classification, operation time, pain score, complications, recurrence, and hospital stay were not reported. Similarly, for the adhesive mesh fixation group, which included 106 participants, no specific details regarding gender distribution, hernia classification, operation time, pain score, complications, recurrence, and hospital stay were provided [18].

Wong et al. (2011) conducted a study on unilateral inguinal hernias, including 26 participants with a mean age of 55.19 years. Among them, 20 were males, and no gender distribution was specified for the remaining participants. The follow-up duration was three months. However, specific details regarding hernia classification, operation time, pain score, complications, recurrence, and hospital stay were not reported. The study did not provide information on the use of adhesive mesh fixation, and the gender distribution for the 30 participants in this group was not provided [19].

In a study by Kim-Fuchs et al. (2011) that utilized the Schumpelick classification, the use of sutures and adhesive mesh fixation for inguinal hernias was investigated. The study included 133 male participants with a mean age of 56.8 years for the sutures group and 131 male participants with a mean age of 55.1 years for the adhesive group. The follow-up duration was 60 months, but specific details regarding pain scores were not reported. The study reported a recurrence rate of 5% for the sutures group and 8% for the adhesive group. The mean hospital stay was 3.39 days for the sutures group and 3.35 days for the adhesive group [20].

Negro et al. (2011) conducted a study involving 171 participants for the sutures group and 349 participants for the adhesive group. The mean age was 55 years for both groups, and the follow-up duration was 12 months. However, specific details regarding hernia classification, operation time, pain score, complications, and recurrence were not reported. The study reported a recurrence rate of 0% for both groups. The mean hospital stay was 1.1 days for the sutures group and 1.5 days for the adhesive group [21].

Paajanen et al. (2011) conducted a study on inguinal hernias without providing specific details on hernia classification, operation time, pain score, complications, recurrence, or hospital stay. The study included 151 participants for both the sutures and adhesive groups. The mean age was 53 years for both groups, and the gender distribution was 135 males and 16 females for the sutures group and 131 males and 20 females for the adhesive group. The follow-up duration was 12 months, but no further information was provided [22].

In a study by Campanelli et al. (2011) focusing on unilateral or bilateral inguinal hernias, 158 male participants were included in both the sutures and adhesive groups. The mean age was 59 years for both groups. The follow-up duration was one month, and the mean operation times were 41.5 minutes for the sutures group and 39.8 minutes for the adhesive group. The pain scores were reported as 1.68 for the sutures group and 1.59 for the adhesive group. Complications were reported in 13 cases for the sutures group and 17 cases for the adhesive group. The recurrence rates were 2% for the sutures group and 1% for the adhesive group. The mean hospital stay was 0.7 days for both groups [23].

In a study by Jani K (2012), the use of sutures and adhesive mesh fixation for hernia repair was investigated without providing specific details on hernia classification, operation time, pain score, complications, recurrence, or hospital stay. The study included 127 participants for the sutures group and 124 participants for the adhesive group. The mean age was 44.8 years for the sutures group and 47.2 years for the adhesive group. The gender distribution was 119 males and eight females for the sutures group and 115 males and nine females for the adhesive group. The follow-up duration was 12 months for the sutures group, but no duration was reported for the adhesive group [24].

Jeyakumar et al. (2018) conducted a study on hernia repair without providing specific details on hernia classification, operation time, complications, recurrence, or hospital stay. The study included 19 participants without specifying the gender distribution. The age range for the participants was 31-60 years. The follow-up duration was six months, and the mean pain score was reported as 2.96. No information was provided regarding the use of adhesive mesh fixation [25].

Shukla et al. (2019) conducted a study without providing specific details on hernia classification, operation time, pain score, complications, recurrence, or hospital stay. The study included 60 participants with a mean age of 45.95 years. The gender distribution was not reported, and the follow-up duration was 12 months. The mean pain score was reported as 5.2 for the sutures group and 2.8 for the adhesive group [26]. Tofigh et al. (2020) conducted a study on hernia repair without providing specific details on hernia classification, pain score, complications, or recurrence. The study included 58 male participants for the sutures group, but no females were included. The mean age was 53.1 years, and the follow-up duration was 12 months. The mean operation time was 73.3 minutes, and the mean hospital stay was 1.6 days. No information was provided regarding the use of adhesive mesh fixation for this group, and further details regarding age, gender distribution, hernia classification, pain score, complications, recurrence, and hospital stay were not reported [27].

Outcome measures in patients with mesh fixation in AWR are shown in Table 2.

**Table 2:** Outcome measure in patients with mesh fixation in abdominal wall reconstruction

Study	Type of surgery	Duration of follow-up (months)	Hernia details	Operation time	Pain score	Complications	Recurrence	Hospital-stay	
Nowobilski et al., 2004	sutures	4.7	Unilateral inguinal hernias - Suture: 33% direct hernia - Glue: 36% direct hernia	42.1	5	0	5	1.75	
	adhesive			40.2	4.1	0	8	1.25	
Hidalgo et al., 2005	sutures	12	Bilateral inguinal hernia - Similar characteristics - No significant difference in their classification	-	-	3	0	-	
	adhesive					2	0		
Testini et al., 2010	sutures	14-60	- Unilateral inguinal hernia	54.5	-	9	0	1.32	
	adhesive			55.1		6	0	1.3	
Wong et al., 2011	sutures	3	Unilateral inguinal hernia - Gilbert classification was	-	1.9	7	-	-	
	adhesive				1.5	4			
Kim-Fuchs et al., 2011	sutures	60	Unilateral inguinal hernia - Schumpelick classification used to define hernia details	79	-	3	5	3.39	
	adhesive			73		5	8	3.35	
Negro et al., 2011	sutures	12	-	64.2	3.2	43	0	1.1	
	adhesive	12		55.6	2.5	39	0	1.5	
Paajanen et al., 2011	sutures	12	inguinal hernia	36	1	6	2	-	
	adhesive			34	1	13	2		
Giampiero et al., 2011	sutures	1	Unilateral or bilateral inguinal hernia	41.5	1.68	13	2	0.7	
	adhesive			39.8	1.59	17	1	0.7	
Jani K, 2012	sutures	12	-	-	0.74	12	0	-	
	adhesive				0.41	8	0		
Jeyakumar et al., 2018	sutures	6	-	52.6	2.96	0	0	-	
	adhesive			41.8	4.88	0	0		
Shukla et al., 2019	sutures	12	-	-	5.2	0	0	-	
	adhesive				2.8	0	0		
Arash et al., 2020	sutures	12	-	73.3	4.99	0	0	1.6	
	adhesive			64.5	4.74	0	0	1.4	

Discussion

Tissue glues have been utilized in surgery for various purposes, such as closing abdominal skin wounds, achieving hemostasis during liver resection, and treating gastro-esophageal bleeding and varices endoscopically. The use of fibrin-based (Tissucol/Tisseel by Baxter Healthcare) and N butyl-2-cyanoacrylate-based adhesives (Glubran 2-GEM Srl) in inguinal hernia surgery was first reported in the mid-1990s. However, concerns persist regarding the long-term outcomes of glue-based fixation techniques. There is a potential risk of mesh migration or rolling due to the decreased strength of the glue over time, which may result in higher recurrence rates or discomfort for the patient [28].

This systematic review indicates that glue mesh fixation (GMF) in open inguinal hernia repair (OIHR) yields results that are statistically comparable to suture mesh fixation (SMF). The recurrence and complication rates for GMF are similar to SMF, and the prevalence of chronic groin pain and length of hospital stay are also comparable. Additionally, GMF offers the advantage of shorter operative time compared to SMF. While postoperative and chronic groin pain tend to favor GMF over SMF, these differences did not reach statistical significance in our study.

The development of chronic groin pain (CGP) following open inguinal hernia repair is influenced by various factors. Pain can arise from nerve resection, nerve compression by sutures, foreign body reaction to the mesh, or tension on muscle fibers. Different strategies have been reported to address postoperative CGP, with varying outcomes and controversies.

One effective approach is the careful identification and preservation of regional nerves during inguinal hernia surgery, which has been shown to reduce the overall incidence of CGP from 21.6% to 5.5%. Another method is the use of lightweight mesh in Lichtenstein hernioplasty, which has demonstrated lower rates of CGP in a recently published meta-analysis of 11 randomized controlled trials. Innovations in hernia surgery include self-gripping meshes with absorbable micro hooks on their surface for tissue fixation, eliminating the need for suture or glue fixation. This technique has generated significant interest in addressing CGP. However, only two randomized controlled trials have been published in this area so far. The Danish DANGROUP study found no difference in acute or chronic pain after open inguinal hernia repair with self-gripping mesh compared to sutures. However, interim results from Kingsnorth et al. have shown improved pain scores in the self-gripping mesh group, indicating potential benefits [29].

Based on the findings of this article, using atraumatic mesh fixation with glue could potentially be an additional measure to reduce the incidence of CGP. It is crucial to perform careful dissection and avoid nerve entrapment during mesh fixation and posterior wall repair, as this can play a significant role in controlling the occurrence of CGP. The authors suggest that a multidirectional approach incorporating a combination of these strategies should be considered to effectively reduce the incidence of CGP in patients undergoing open inguinal hernia repair. It is important to note that focusing solely on changing the mesh fixation technique may not be sufficient [30].

Study limitations

The present review has several limitations that need to be acknowledged. Firstly, there were significant variations in the inclusion and exclusion criteria among the trials included in this review, including the recruitment of patients with unilateral or bilateral inguinal hernias. The lack of sub-classification of inguinal hernias into direct and indirect types during patient selection could have a notable impact on the results [31]. Furthermore, there was considerable variation in the administration of glue among the trials, with some using dots of glue in place of sutures, while others applied a thin film of glue for mesh adhesion. Similarly, the method of hernia repair varied, with two studies incorporating hernia plugs in addition to a mesh layer on the posterior wall, which may influence complication and postoperative pain rates [32].

The definitions of "chronic groin pain" and the measurement scales used to assess postoperative pain also differed among the trials. Moreover, the relatively small sample sizes in some of the randomized controlled trials included in this review may have limited their ability to detect small differences in outcomes. The duration of follow-up varied greatly among the included studies, ranging from three months to 60 months, with the majority having a follow-up period of one year or less. This limited follow-up duration may be insufficient for accurately determining recurrence and chronic groin pain rates [33]. Important variables such as foreign body sensation, groin stiffness, and decreased groin compliance should have been considered since displaced or rolled-up mesh is more likely to cause these symptoms [34].

Additionally, before glue fixation can be universally adopted, a cost-benefit comparison is necessary to assess the economic viability of using glue compared to suture fixation. It should be noted that fibrin glue typically costs approximately 100 Euros per milliliter, whereas sutures cost around 10 Euros. These limitations highlight the need for further research with standardized criteria, longer follow-up periods, and comprehensive assessment of relevant variables to provide more robust evidence on the effectiveness and feasibility of glue fixation in inguinal hernia repair.

## Conclusions

Based on the summated outcome of this review, it can be concluded that atraumatic mesh fixation with glue in open inguinal hernia repair shows promise as a potential measure to reduce the incidence of chronic groin pain (CGP). However, it is important to consider the limitations of the included trials, such as variations in patient selection criteria, glue administration techniques, and hernia repair methods. Additionally, the definitions of CGP and measurement scales for postoperative pain varied among the trials. The relatively small sample sizes and limited follow-up durations in some studies may have affected the ability to detect subtle differences in outcomes and accurately assess long-term recurrence and chronic groin pain rates. Furthermore, the economic implications of adopting glue fixation, considering the higher cost of fibrin glue compared to sutures, need to be carefully evaluated. Therefore, while the findings suggest the potential benefits of atraumatic mesh fixation with glue, further research with standardized criteria, longer follow-up periods, and comprehensive assessment of relevant variables is necessary to establish its effectiveness and feasibility in reducing CGP in open inguinal hernia repair.
